# The translocator protein 18kDa ligand etifoxine in the treatment of depressive disorders—a double-blind, randomized, placebo-controlled proof-of-concept study

**DOI:** 10.1186/s13063-024-08120-x

**Published:** 2024-04-22

**Authors:** Lisa-Marie Brunner, Marco Riebel, Simon Wein, Michael Koller, Florian Zeman, Gunnar Huppertz, Tanja Emmer, Yvonne Eberhardt, Jens Schwarzbach, Rainer Rupprecht, Caroline Nothdurfter

**Affiliations:** 1 Department of Psychiatry and Psychotherapy, University of Regensburg, Regensburg, Germany; 2 Center for Clinical Studies, University Hospital of Regensburg, Regensburg, Germany

**Keywords:** Randomized controlled trial, TSPO, Etifoxine, Depression, Neurosteroids, Functional MRI, Microbiome

## Abstract

**Background:**

Recent developments suggest that neurosteroids may achieve rapid antidepressant effects. As such, neurosteroidogenesis mediated by the translocator protein 18 kDa (TSPO) might constitute a promising option for the treatment of depression. Therefore, the current clinical trial aims to get the first evidence of whether TPSO ligands promote rapid antidepressant effects. Furthermore, we study which mechanisms of action, e.g., modulation of distinct neuronal networks, neurosteroidogenesis, endocrinological mechanisms, TSPO expression or microbiome composition, contribute to their putative antidepressant effects.

**Methods:**

This is a randomized, placebo-controlled, double-blind single-center trial of 2-week treatment with the TSPO ligand etifoxine versus placebo in depressive patients. Main eligibility criteria: male or female individuals aged 18 to 65 years with unipolar/bipolar depressive disorder with no other psychiatric main diagnosis or acute neurological/somatic disorder or drug/alcohol dependence during their lifetime.

The primary endpoint is the time point at which 50% of the maximal effect has occurred (ET50) estimated by the scores of the Hamilton Depression Scale (HAMD-21). A total of 20 patients per group are needed to detect changes of therapeutic efficacy about 5% and changes of ET50 about 10% with a power of 70%. Assuming a drop-out rate of 10–20%, 50 patients will be randomized in total. The study will be conducted at the Department of Psychiatry and Psychotherapy of the University of Regensburg.

**Discussion:**

This study will provide a first proof-of-concept on the potential of the TSPO ligand etifoxine in the treatment of depressive disorders.

**Trial registration:**

Clinical Trials Register (EudraCT number: 2021-006773-38, registration date: 14 September 2022) and German Register of Clinical Studies (DRKS number: DRKS00031099, registration date: 23 January 2023).

## Administrative information

Note: the numbers in curly brackets in this protocol refer to SPIRIT checklist item numbers. The order of the items has been modified to group similar items (see http://www.equator-network.org/reporting-guidelines/spirit-2013-statement-defining-standard-protocol-items-for-clinical-trials/).

**Table:** 

Title {1}	The translocator protein 18kDa ligand etifoxine in the treatment of depressive disorders—a double-blind, randomized, placebo-controlled proof-of-concept study
Trial registration {2a and 2b}.	Clinical Trials Register (EudraCT number: 2021–006773-38; https://www.clinicaltrialsregister.eu/ctr-search/trial/2021-006773-38/DE) and German Register of Clinical Studies (DRKS number: DRKS00031099; https://drks.de/search/de/trial/DRKS00031099)
Protocol version {3}	Version 4 of 7–7-2023
Funding {4}	This work is supported by the German Research Foundation (Deutsche Forschungsgemeinschaft) (DFG), project number 422179811, to CN, JS, and RR and within the framework of FOR2858.
Author details {5a}	Lisa-Marie Brunner: Department of Psychiatry and Psychotherapy, University of Regensburg, Germany Marco Riebel: Department of Psychiatry and Psychotherapy, University of Regensburg, Germany Simon Wein: Department of Psychiatry and Psychotherapy, University of Regensburg, Germany Michael Koller: Center for Clinical Studies, University Hospital of Regensburg, Germany Florian Zeman: Center for Clinical Studies, University Hospital of Regensburg, Germany Gunnar Huppertz: Center for Clinical Studies, University Hospital of Regensburg, Germany Tanja Emmer: Center for Clinical Studies, University Hospital of Regensburg, Germany Yvonne Eberhardt: Center for Clinical Studies, University Hospital of Regensburg, Germany Jens Schwarzbach: Department of Psychiatry and Psychotherapy, University of Regensburg, Germany Rainer Rupprecht: Department of Psychiatry and Psychotherapy, University of Regensburg, Germany Caroline Nothdurfter: Department of Psychiatry and Psychotherapy, University of Regensburg, Germany
Name and contact information for the trial sponsor {5b}	medbo, medical facilities of the district Oberpfalz – KU (institution under public law; represented by Helmut Hausner (head of the clinic), helmut.hausner@medbo.de
Role of sponsor {5c}	The sponsor carries the medico-legal responsibility of the trial. This is an investigator initiated clinical trial. Therefore, the funders played no role in the design of the study and collection, analysis, and interpretation of data and in writing the manuscript.

## Introduction

### Background and rationale {6a}

Neurosteroidogenesis mediated by the translocator protein 18 kDa (TSPO) [1–4] plays an important role for psychiatric disorders and their treatment. Meanwhile, numerous studies have shown altered expression of TSPO in psychiatric disorders [5, 6]. These studies targeted either the expression of TSPO mRNA in peripheral mononuclear cells, the binding characteristics of the TSPO ligand PK11195 to platelet membranes, or protein expression in thrombocytes [7–9]. Various positron emission tomography (PET) studies reported increased TSPO expression in depression [10, 11]. It has even been suggested that TSPO PET imaging may predict the clinical response to celecoxib treatment in major depression [12]. Moreover, in depression with cognitive impairment upregulation of TSPO labeling has been reported using PET [11]. Gene variants such as the rs6971 TSPO polymorphism, which affects ligand binding and cholesterol uptake, were found to be linked to both bipolar disorder in general and diurnal cortisol rhythm in bipolar disorder [13, 14]. Therefore, it should be considered in clinical trials when assessing TSPO binding or function.

Various TSPO ligands have been shown to exert acute anxiolytic/anti-conflict activity in rodents [2, 6, 15]. In a translational study, the selective TSPO ligand XBD173 enhanced GABAergic neurotransmission in brain slices via the induction of neurosteroidogenesis and effectively reduced the number of pharmacologically induced panic attacks in rodents in the absence of sedation [15]. Moreover, XBD173 displayed anti-panic and anxiolytic efficacy in humans using an experimental anxiety paradigm involving a challenge with cholecystokinin tetrapeptide. Noteworthy, sedation and withdrawal symptoms occurring for benzodiazepines were absent in the XBD173-treated subjects after 7 days of treatment. A recent animal study suggested that the TSPO ligand AC-5216 (XBD173) may exert rapid antidepressant and memory-enhancing effects [16]. The capability of TSPO ligands to exert anxiolytic and antidepressant effects has also been demonstrated for other novel TSPO ligands such as YL-IPAo8 in a rat model of postpartum depression [17] or the antagonistic ligand ONO-2952 [18]. As such, TSPO may represent a promising target for the development of fast-acting anxiolytics and anti-depressants with a favorable side effect profile.

Currently, the only clinically available TSPO ligand is etifoxine (Stresam, Biocodex), which has been approved for the treatment of anxiety disorders in France. Etifoxine has a dual mode of action, as it targets TSPO but also directly α2 and α3 containing GABA(A) receptors [19]. Initial clinical trials with etifoxine have provided the first evidence for a clinical anxiolytic effect of etifoxine showing comparable efficacy to the benzodiazepine lorazepam in patients suffering from adjustment disorders with anxiety [20]. The anxiolytic effects of etifoxine are comparable to clonazepam and have recently been confirmed in a randomized controlled double-blind clinical trial in patients with anxiety disorder [21].

GABAergic modulators such as benzodiazepines are known to inhibit long-term synaptic potentiation necessary for memory formation [22]. Using functional magnetic resonance imaging (fMRI) and an acute challenge with the benzodiazepine lorazepam in healthy participants, it was found that blood oxygenation level-dependent (BOLD) responses in the amygdala to novel stimuli were attenuated, which is in line with the known risk of benzodiazepines causing anterograde amnesia [23]. However, it is unknown whether or to which degree TSPO ligands such as etifoxine, which may also target extrasynaptic GABA(A) receptors by means of endogenous neurosteroidogenesis [19, 24], affect learning and memory. To this aim, we developed an event-related version of the face-name-association (FNA) task employed by Sperling et al. [23]. This task allows us to trace the neural signature of novel stimuli during memory acquisition on a trial-by-trial basis and to characterize the efficiency of learning by the slope BOLD amplitude as a function of a number of stimulus repetitions.

Antidepressant treatment is related to neural, cognitive, and emotional changes in patients. It has been demonstrated that fMRI in combination with a multivariate technique, a so-called representational similarity analysis (RSA), is able to assess the neural representation of an individualized relational affective space [25]. Recently, it could be shown how RSA can be employed in psychiatry to assess the commonalities in cognitive-emotional and neural changes in the context of interventions in mental disorders [26].

Because also bacteria express TSPO and bacterial TSPO shares structural and functional characteristics with mammalian TSPO [27], TSPO ligands may also affect microbiome composition. So far, it could be shown that short-term etifoxine intervention reduces the abundance of a few bacterial species, which are currently seen as beneficial components of a healthy intestinal microbiome. This reduction may be mainly related to elevated endogenous neurosteroids [28].

Another promising approach is the direct application of neurosteroids such as the intravenous formulation of brexanolone [29] or the oral administration of zuranolone (SAGE-217) [30]. Intriguingly, these compounds may exert quite rapid antidepressant effects. While brexanolone and zuranolone have recently been approved for the treatment of postpartum depression by the FDA, the antidepressant properties of zuranolone are currently being investigated in a series of multicenter studies and still await approval by the FDA. These recent developments and the discrete neuroinflammation reported in depression have promoted the idea that TSPO ligands may constitute an attractive alternative to exogenous neurosteroids in the treatment of depression [6]. Therefore, the current clinical trial aims to get the first evidence of whether the TPSO ligand etifoxine may confer rapid antidepressant effects and which mechanisms of action, e.g., modulation of distinct neuronal networks, neurosteroidogenesis, HPA axis activity, TSPO expression and of microbiome composition, contribute to its putative antidepressant effects.

### Objectives {7}

#### General objective

Within this clinical trial, we will investigate whether the administration of the TSPO ligand etifoxine in addition to treatment as usual (TAU) changes the time course or the extent of the therapeutic response in patients with severe unipolar/bipolar depression. Besides clinical effects as well as physiological and endocrine markers, we will assess the impact of the treatment on neuronal networks. Furthermore, changes of microbiome composition will be studied.

#### Primary objective

The primary objective of the trial is to investigate the effects of add-on treatment with etifoxine on clinical symptoms in patients with severe unipolar/bipolar depressive disorder. Clinical symptoms will be assessed using the Hamilton Depression Scale (HAMD-21) [31] up to day 15 after the start of the treatment.

- Does add-on treatment with the TSPO ligand etifoxine in addition to TAU for 14 days fasten the treatment response (decrease of ET50; ET50 = time point at which 50% of the maximal effect has occurred) in comparison to placebo?

#### Secondary objectives

Does add-on treatment with etifoxine compared to placebo in addition to TAU in depressive patients lead to.

- A decrease of the Emax (= maximum effect which can be expected from the drug)?
- A reduction of the HAMD-21 score on day 15?
- Changes of the synthesis of neurosteroids, TSPO expression or activity of the hypothalamic–pituitary–adrenal axis?
- Changes in cognitive functions like memory or emotional processing assessed with a neuropsychological test battery?
- Changes of functional neuronal networks and cognitive functions assessed using fMRI applying resting state measurements and two tasks ((representational similarity analysis) RSA and face-name-association (FNA))?
- Changes of the microbiome composition?
- A relapse or withdrawal symptoms after discontinuation of the intake?

### Trial design {8}

This is a single-center, randomized, parallel-group, placebo-controlled, double-blind investigator-initiated superiority clinical trial (IIT) with two arms. Eligible patients will receive either etifoxine or placebo for a period of 14 days. The patient allocation ratio is 1:1. Measurements will be taken up to 29 days after the start of the study treatment. The trial will be conducted with 50 patients suffering from unipolar/bipolar depressive disorder.

## Methods: participants, interventions, and outcomes

### Study setting {9}

The trial will be performed at the Department of Psychiatry and Psychotherapy of the University of Regensburg in Germany. It is an acute psychiatric clinic providing the highest level of care with 475 inpatient treatment places and outpatient care for psychiatric patients. Affective disorders account for the second largest number of inpatient admissions at the clinic meaning that a correspondingly large number of patients can be expected.

### Eligibility criteria {10}

In total, 50 patients will be included in the clinical trial if they meet the study criteria.

#### Inclusion criteria

- Inpatient treatment at the Department of Psychiatry and Psychotherapy, University Regensburg
- Male and female patients in the age between 18 and 65 years
- Voluntary admission to the hospital independently from the trial
- Diagnosis of unipolar (ICD-10: F32, F33) or bipolar depression (ICD-10: F31.3–5)
- HAMD-21 score > 18
- Ability to conceive nature, meaning, and consequences of participation in the clinical trial and to understand and implement the explanations and instructions concerning the study
- Written informed consent after the trial has been comprehensively explained
- Indication for pharmacological treatment independently from the trial
- Willingness to forgo the consumption of alcohol during participation in the study
- Women of Childbearing Potential (WOCBP) need a negative pregnancy test (serum β-hCG = serum human chorionic gonadotropin) at inclusion and need to be willing to use reliable contraception during the study (e.g., oral contraceptives, hormone-containing intrauterine coils, dermal or injectable contraceptives with long-term effects, tubal ligation). WOCBP are defined as women after menarche, which are not post-menopausal (at least 12 months no menstruation) and which did not undergo a documented hysterectomy, bilateral salpingectomy, or bilateral oophorectomy.
- Willingness to forgo to drive a car or to operate heavy machines
- Patients with partners in a reproductive age must be willing to use appropriate contraceptives (Pearl-Index < 1%) for the duration of the study
- During pregnancy of the partner the patients need to use a condom during sexual intercourse

#### Exclusion criteria

- Diagnosis of a comorbid mental disorder like schizophrenia, addiction disorders according to ICD-10, or presence of another psychiatric main diagnosis in accordance with ICD-11 diagnosed using the Mini-International Neuropsychiatric Interview (M.I.N.I.) [32]
- Diagnosis of a severe somatic or neurological disease
- Acute suicidality
- Contraindications of the IMP: myasthenia, state of shock, severely impaired liver and/or renal function
- Contraindications against the implementation of functional Imaging (pacemaker, metal implants, tattoos in the head/neck area)
- Permanent treatment with 5alpha-reductase-inhibitors, pregabalin, or gabapentin over 2 weeks prior to participation in the study
- Heart rate (HR) < 45 or > 110 bpm
- Clinically relevant impairments in ECG
- Blood pressure: systolic < 90 or > 165 mm Hg, diastolic < 50 or > 95 mm HG
- Body temperature < 35 °C or > 37.5 °C
- BMI < 19 bzw. > 35
- Abnormal laboratory parameters of clinical relevance before study inclusion: Excess of thresholds: GPT, GOT and γ-GT above 20%, creatinine up to 0.2 mg/dL above age-adapted threshold; excess of the normal range more than twice as much of the upper standard or underrun of more than half of the lower standard for the other laboratory parameters (erythrocytes, leucocytes, thrombocytes, hemoglobin, hematocrit, MCH, MCHC, MCV, lymphocytes, monocytes, eosinophils, basophils, neutrophils, natrium, potassium, calcium, transferrin, ferritin, urea, uric acid, sober glucose, overall protein, triglycerides, cholesterol, HDL, LDL, C-reactive protein (CRP), bilirubin, TSH, free triiodthyronine (fT3), free thyroxine (fT4), Quick, PTT, HbA1c)
- Pregnancy or nursing period
- Abuse of alcohol or drugs within the last 12 months before the inclusion screening diagnosed using the M.I.N.I.
- Dependence of alcohol or drugs in the medical history diagnosed using the M.I.N.I.
- Known allergy or hypersensitivity against etifoxine hydrochloride or one of the other components (talc, docusate sodium, sodium benzoate, preagglutinated starch, microcrystalline cellulose, Lactose Monohydrate, Magnesium stearate (Ph. Eur.), highly-dispersed silicon dioxide, titanium dioxide, indigotine, erythrosine)
- Galactose intolerance, lack of lactose, glucose-galactose malabsorption
- Celiac disease, non-celiac-non-wheat allergic-wheat sensitivity (NCHS)
- Positive drug screening (amphetamines, cannabis, opiates, cocaine, ethyl glucuronide, ethanol, fentanyl, pregabalin, buprenorphine, methadone)
- Concurrent participation in another clinical trial according to AMG

### Who will take informed consent? {26a}

Before participation in the clinical trial, suitable patients are informed in detail about the clinical trial, its purpose, procedure, the investigational product, and possible risks both orally by a medical member or a psychologist of the trial team and in writing by the patient information leaflet. They will be notified about the voluntariness of participation in the study without any disadvantages for further clinical care in case of discontinuation. Afterwards, the patient is given at least 24 h to think about participating in the clinical trial. In case of agreement to join the trial patients will be asked to provide written consent.

### Additional consent provisions for collection and use of participant data and biological specimens {26b}

Not applicable, there are no ancillary studies planned. All information is provided within the patient information leaflet.

## Interventions

### Explanation for the choice of comparators {6b}

Patients in the control group will receive placebo capsules in addition to TAU. Since all study patients are allowed to be further treated with TAU, there are no ethical concerns of this control condition.

### Intervention description {11a}

During the treatment phase, the patients receive either 200 mg etifoxine/day (100–0–100 mg) or placebo treatment as an add-on to TAU for 14 days. During the treatment phase, the patients should take the medication orally (capsules) in two single doses (consisting of 2 capsules of 50 mg each) at 8:00 am and 6:00 pm.

The study medication will be administered to the patients by the nursing staff of the respective ward on all days at the two respective time points. The study medication is administered to the nursing staff by the medical members of the study group or the study assistants. The study medication may only be administered in accordance with the protocol guidelines.

### Criteria for discontinuing or modifying allocated interventions {11b}

Patients can end participation in the trial at any time without any consequences for further care. They must be withdrawn from the trial when judged necessary by the investigator, as for safety concerns, severe non-compliance, incorrect inclusion, or new development of an exclusion criterion. The data that have been collected up to that time point will be included in the analysis.

### Strategies to improve adherence to interventions {11c}

Some of the measurements taken in this trial can be integrated into the clinical routine. Both, the incidence of serious adverse reactions to the investigational product and risks of harm by any of the measurements are low. In the first week of treatment, patients will have daily contact to one of the investigators. Also afterwards, there will be regular communication with the patients to improve compliance and minimize the risk of drop-outs.

### Relevant concomitant care permitted or prohibited during the trial {11d}

The additional administration of benzodiazepines (lorazepam up to 3 mg/day) should only take place in cases of clinical necessity. On the days before the fMRI scans, the concomitant medication should not be administered if possible.

### Provisions for post-trial care {30}

After completion of the clinical trial patients continue to be treated by the ward doctors, provided they still require inpatient treatment. Any harm that occurs during the course of participation will be covered by trial insurance.

### Outcomes {12}

#### Self-ratings

To assess the course of the clinical symptoms and respective effects of the treatment several psychometric instruments will be applied. Using the Patient Health Questionnaire 9 (PHQ-9) [33] and the Beck-Depression-Inventory (BDI) [34] depressive symptoms will be rated. Visual analog scales (VAS) [35] will be used to check for further symptoms associated to depression (depressive mood, anxiousness, lack of drive, and somatic issues) as well as an estimation of the general state. Using the Stanford Sleepiness Scale (SSS) [36], we will check for daily sleepiness. Possible symptoms related to withdrawal or drug dependence will be assessed using the Benzodiazepine Hypnotics Withdrawal Symptom Scale (BHWSS) [37] and the Benzodiazepine Dependence Self-Report Questionnaire (BENDEP-SRQ) [38].

#### External ratings

All external ratings will be conducted by experienced members of the trial team (physicians, psychologists). The severity of depressive symptoms will be assessed by the Hamilton-Depression-Scale (HAMD-21) as well as the Montgomery–Åsberg Depression Rating Scale (MADRS) [39]. Anxiety symptoms will be assessed using the Hamilton-Anxiety-Scale (HAMA) [40]. Additionally, we will determine the risk of suicidality with the Columbia Suicide Severity Rating Scale (C-SSRS) [41].

#### Functional magnetic resonance imaging

We will perform (f)MRI measurements at a Siemens 3 Tesla MRI scanner. Each session will consist of one resting-state measurement and two task-based scans.

##### Resting-state measurements

Using task-free resting-state fMRI, we plan to identify so-called functional networks, which reflect the integration of information processing in different brain regions underlying complex behavior [42]. Therefore, patients are asked to lie still in the MRI scanner for 22 min, close their eyes, and not to fall asleep.

##### Task 1: cognitive and neuronal representations

This task consists of a behavioral and an imaging part. In the behavioral part, patients are asked to arrange emotional terms on a computer screen in a 2-dimensional space using “drag-and-drop.” In this so-called “ARENA method,” the distances between the terms reflect their dissimilarity [43]. In the imaging part, which was adapted from Thornton et al. [44], patients are presented with two different statements (e.g., “I am happy,” “I am content”) of the same emotion class, e.g., “Joy,” together with the class name above the statements. Patients are asked to press a button to indicate which of these statements better represents the emotional category for them. RSA can be used to search for brain regions that show neuronal distance patterns similar to the behavioral distance matrices.

##### Task 2: face-name-associations

In this task, patients are first presented with two pairs of faces and written names in an event-based paradigm with the instruction to learn these two associations. Afterwards, the previously shown face-name pairs (old) are presented alternately with new face-name pairs (new). The aim for the patients is to learn as many face-name pairs as possible for a subsequent memory test. This task allows us to measure the hypothesized indirect effect of etifoxine on the GABAergic system. An attenuation of the BOLD response to novel stimuli was shown after acute administration of the benzodiazepine lorazepam [23]. In addition to the design of Sperling et al., a measurement is made during the acquisition phase, which makes it possible to check whether the administration of etifoxine leads to a flattening of the so-called repetition suppression [4] compared to placebo and whether this results in reduced performance in the subsequent memory test.

#### Cambridge Neuropsychological Test Automated Battery (CANTAB)

The Cambridge Neuropsychological Test Automated Battery (CANTAB, Cambridge Cognition Lt., Cambridge, UK) is a computer-based procedure that contains a total of 25 tests to record cognitive abilities. These can be used to assess various axes of cognition, such as sustained attention, the ability to recognize visual correspondences and basic emotions, and memory performance. The total completion time is approximately 40 min. The following subtests are used in this clinical trial:

- Rapid Visual Information Processing (7 min): measurement of sustained attention
- One Touch Stockings of Cambridge (10 min): assessment of executive function via the subdomains of spatial planning and working memory
- Spatial Working Memory (4 min): assessment of executive function and strategic approach/working memory
- Delayed Match to Sample (7 min): testing the ability to recognize visual matches and short-term recognition of visual stimuli
- Emotion Recognition Task (6–10 min): assessment of the ability to recognize the 6 different basic emotions using morphed facial expressions on a continuum

#### Blood samples

During the course of the clinical trial, venous blood is taken from the patients on the one hand to screen for safety parameters (screening or safety blood) on the other to test secondary endpoints (study blood). The following blood parameters are collected in each case:

##### Screening or safety blood (sober)

Erythrocytes, leukocytes, thrombocytes, hemoglobin, hematocrit, MCH, MCHC, MCV, lymphocytes, monocytes, eosinophils, basophils, neutrophils, sodium, potassium, calcium, transferrin, ferritin, creatinine, urea, uric acid, fasting glucose, total protein, bilirubin, triglycerides, cholesterol, HDL, LDL, GPT, GOT, γ-GT, C-reactive protein (CRP), TSH, free triiodothyronine (fT3), free thyroxine (fT4).

Additionally, during screening: Quick, PTT, HbA1c; for WOCBP: β-HCG.

##### Study blood

Platelets (determination of TSPO expression by Western blot), serum (creation of a neurosteroid profile using tandem mass spectrometry; cortisol (ELISA), whole blood (genotyping regarding the TSPO gene polymorphism rs6971).

#### Cortisol awakening response

To determine the cortisol awakening response (CAR) [45] saliva samples are taken from the patients using salivettes (chewing on cellulose rolls) (Sarstedt AG & Co., Nümbrecht, Germany) directly as well as 30 and 60 min after waking up. The saliva samples are stored for a short time at room temperature and in a freezer at – 20 °C until biochemical analysis.

#### Microbiome composition

To determine the bacterial composition of the human microbiome in depressive patients and its changes during the add-on treatment with etifoxine or placebo, stool samples from the patients will be analyzed. The complete genome will be sequenced using DNA. For this purpose, a stool sample is preserved in a sample container suitable for microbiome analysis and deep-frozen at – 80 °C for later analysis of the composition of the colon microbiome.

### Participant timeline {13}

#### Screening (D-1)

If participation is desired after the informed consent discussion, an appointment for the screening is made, which should take place maximum 1 week after the discussion.

After checking the dated signature of the patient information, the screening is started with the first blood sample (screening blood) and the urine sample for drug screening. For Women of Childbearing Potential (WOCBP), an additional pregnancy test will be carried out by assessing ß-HCG level in screening blood. A detailed psychiatric and somatic anamnesis is taken from all patients to rule out any mental comorbidities and physical illnesses. The Mini-International Neuropsychiatric Interview (M.I.N.I.) [32] will be used. In addition, an orienting general physical and special neurological examination is carried out as well as the measurement of blood pressure/pulse and ECG and the recording of body weight/height and body temperature. Furthermore, all other inclusion/exclusion criteria, concomitant diseases, and the use of stimulants are assessed.

#### Baseline (D0)

On the baseline day, the patients are asked to collect saliva samples using salivettes immediately and 30 and 60 min after waking up to determine the cortisol awakening response (CAR). Study blood and stool samples are also taken in the morning. In addition, vital signs such as weight are measured. Further, the self-evaluation questionnaires are handed out to the patients for completion and the external assessment ratings are carried out. The first measurement with the CANTAB test battery and the ARENA task also take place on this day. These two experimental paradigms can also be carried out on screening day T-1 after all inclusion/exclusion criteria have been checked. In the afternoon, the MRI session will take place at the research MRI scanner on the clinic premises.

#### Days 1 to 8 of treatment (D1 to D8)

Drug treatment begins one day after the baseline. From day 1 of treatment, patients receive the randomly assigned medication (etifoxine or placebo) daily from the nursing staff of the respective ward. The study medication should be taken at 8:00 am and 6:00 pm. On days 1 to 8 of treatment, the HAMD-21 and the MADRS are carried out daily with the patients, and the PHQ-9 and the VAS are given for completion. Possible AEs are recorded on days 1, 8, 15, 22, and 29. On day 8 of treatment, all other self-assessment and external assessment instruments are presented again.

#### Days 9 to 14 of treatment (D9 to 14)

Only the study medication is taken on these days. No examinations are carried out. AEs are not specifically queried but are recorded if they occur.

#### Day 15 after baseline (D15)

On day 15 after the start of treatment patients provide further saliva samples to determine the CAR. Also in the morning, the safety and study blood samples are taken, and the stool sample should be delivered. In addition, vital signs such as weight are determined. Blood pressure/pulse, ECG, and body temperature are also measured. The self-assessment questionnaires are presented to the patients for completion and the external assessment ratings are carried out. Afterwards, the second measurement with the CANTAB test battery and the ARENA method takes place. In the afternoon, the second MRI session is conducted.

#### Day 22 after baseline (D22)

On day 22, after the start of treatment, patients are asked to complete the self-assessment questionnaires, and the external assessment ratings are carried out.

#### Day 29 after baseline (D29)

At the final visit on day 29 after the start of treatment, the safety and study blood samples are taken, and the stool sample is submitted. In addition, vital signs are measured, and a physical examination is carried out. Patients are given the self-assessment questionnaires and the external assessment ratings are carried out. In the afternoon, the last MRI session takes place.

An overview of the study plan including all measures on safety as well as efficacy is given as a schedule of activities in Table 1.

**Table 1:** Schedule of activities

Study day	D − 1	D0	D1	D2	D3	D4	D5	T6	D7	D8	D14	D15	D22	D29
	Screening	Baseline	Treatment phase									Follow-up		
Screening/safety blood	X											X		X
Drug screening urine	X													
Pregnancy test	X^A^													
Medical history	X													
MINI	X													
Physical/neurological examination	X													X
Vital parameters	X											X		X
Body weight	X											X		X
Concomitant medication/(concomitant diseases)	X											X		
HAMD-21, PHQ-9, VAS, MADRS		X	X	X	X	X	X	X	X	X		X	X	X
HAM-A, C-SSRS, BDI, SSS, BHWSS, BENDEP-SRW		X								X		X	X	X
Study medication			X^B^									X^C^		
Assessment of adverse events			X							X		X	X	X
Study blood		X										X		X
CANTAB, ARENA^D^		X										X		
fMRI		X										X		X
Cortisol Awakening Response		X										X		
Microbiome		X										X		X

^A^For WOCBP

^B^200 mg/day etifoxine or placebo

^C^Treatment according to clinical judgment

^D^These two experimental paradigms can also be performed on D-1 after checking all inclusion/exclusion criteria

### Sample size {14}

The calculation of the sensitivity of the planned analyses is based on recent work, which investigated the effects of treatment with SAGE-271 (zuranolone) compared to placebo in patients with the depressive disorder [30]. We used least squares approximation to estimate the parameters of an Emax model [46] considering the scores of the HAMD-21 reported by Gunduz-Bruce and colleagues. This resulted in Emax TAU + PLC = 0.586 and ET50 TAU + PLC = 4.224. This means that comparable TAU with an additional placebo would lead to a reduction of the HAMD-21 scores of 58.6% and that half of this decrease would be reached after slightly more than four days.

Montecarlo simulations of an Emax model based on the mentioned parameter estimations for TAU and on the reported standard deviation of the HAMD-21 scores with s = 2.6 in the study of Gunduz-Bruce et al. [30] show that a sample size of 20 patients per group (TAU + placebo, TAU + etifoxine) is sufficient to detect chances of therapeutic efficacy (Emax) of about 5% and changes of the response latency (ET50) of about 10% with a statistical power of 70%. Assuming a drop-out rate of 10–20% we plan to recruit in total of 50 patients (25 per group).

### Recruitment {15}

We closely communicate with the central occupancy management of the hospital to be pre-informed about potential candidates and to not miss any admission throughout all the wards. Furthermore, we directly informed resident psychiatrists in the surroundings about the trial, placed advertisements with study information on the hospital homepage, and distributed leaflets at relevant locations.

## Assignment of interventions: allocation

### Sequence generation {16a}

The randomization list was set up blockwise by a biometrician of the Center for Clinical Studies, University of Regensburg, using the software SAS 9.4. Patients will be randomly assigned to receive either etifoxine or placebo with an allocation ratio of 1:1.

### Concealment mechanism {16b}

The randomization list was directly sent to the pharmacy of the University Hospital of Erlangen, which is responsible for the production of the study medication as well as the respective documents. This list will not be accessible for any member of the study team during the duration of the trial.

The investigational product and placebo will be repackaged and blinded by the pharmacy of the University Hospital of Erlangen to ensure that the compounds are identical with respect to appearance, packaging, and lablling. All patients receive the same number of capsules.

### Implementation {16c}

All patients who give their informed consent for participation in the trial and who fulfil the inclusion criteria will be randomized. Patients will be randomized in a sequential manner following the order of their inclusion.

## Assignment of interventions: blinding

### Who will be blinded {17a}

The study medication will be administered in a double-blind manner. This means that neither patients nor any member of the study team knows which treatment arm the patients are allocated to. All statistical analyses will be conducted under maintenance of the blinding.

### Procedure for unblinding if needed {17b}

Emergency envelopes for unblinding prepared by the pharmacy are kept locked in the research ward. In the event of an emergency, it is ensured that the trained ward staff has access to those envelopes at all times. In such a case, the sponsor must be informed on the next working day. A written justification will be submitted subsequently in this case. After unblinding, the clinical trial must be terminated for the respective patient.

## Data collection and management

### Plans for assessment and collection of outcomes {18a}

The primary outcome parameter is ET50 estimated using the Hamilton Depression Scale (HAMD-21) scores collected on baseline day and on days 1, 2, 3, 4, 5, 6, 7, 8, 15, 22, and 29 of treatment. To guarantee good quality of the assessment, we have designated a fixed team of raters that were internally trained by experienced psychiatrists with a focus on high inter-rater consistency. Additionally, all ratings for one patient will consistently be performed by the same rater. For a description of all other outcome parameters see {12}.

A web-based, electronic Case Report Form (eCRF) within a validated database management system is used for the database-supported recording of study data. This system enables consistent data storage and quality control. Qualified entry personnel are given access to the eCRF by assigning personal login data. A medical investigator confirms timely with an electronic signature that the study data was entered into the database completely and correctly.

### Plans to promote participant retention and complete follow-up {18b}

In case patients are discharged before the end of the study, there is the option to accomplish the measurements on days 22 and 29 in an outpatient setting. To at least compensate for the effort a little, patients receive monetary incentives of about 150 € as approved by the ethics committee for the completeness of data collection. Overall, the study team has a good track record in enrolling and following participants in previous trials.

### Data management {19}

Data management will be performed by the Center for Clinical Studies, University of Regensburg according to a data management plan. An electronic Case Report Form (Castor, Netherlands) will be used to record all data that are relevant for the analysis of the efficacy and safety data.

### Confidentiality {27}

Patients will receive a study code under which pseudonymized scientific data will be acquired and stored. Clinical Data will be documented in electronic case report forms (eCRFs), which will only refer to the study code. Imaging data will be acquired with the minimum subject identifiers needed for a safe operation of the MRI scanner (study code, weight, height, gender, year of birth).

To protect the confidentiality of personal study-related data we keep the general and study-specific source data at the trial site. The monitor who views the source data and the patient-based eCRFs may only do so to the extent required by her task and is obliged to maintain confidentiality. All documents that leave the trial site contain only pseudonymized patient data.

### Plans for collection, laboratory evaluation, and storage of biological specimens for genetic or molecular analysis in this trial/future use {33}

Using blood samples from the baseline day, we will determine the presence of the TSPO gene polymorphism rs6971. Patients are fully informed about that by the information leaflet. There are no further genetic analyses or storage in repositories planned.

## Statistical methods

### Statistical methods for primary and secondary outcomes {20a}

Statistical analyses will be conducted by the Center for Clinical Studies, University of Regensburg in collaboration with the Biomedical Imaging group of the Department of Psychiatry and Psychotherapy, University of Regensburg. We will use SAS (version 9.4 or higher), Matlab (version 2021a or higher), R (R Core Team, 2020), and scikit-learn (1.3.2 or higher) for the analyses.

Baseline characteristics and demographic data will be presented descriptively for all patients and separately for the two treatment groups. Categorical variables will be reported by absolute and relative frequencies, interval scaled data using descriptive values like mean, standard deviation, median, interquartile range, minimum, and maximum.

Primary endpoint: Using the Nelder-Mead Simplex method (implemented in Matlab) we will conduct a nonlinear minimization of the square deviation of the HAMD-21 scores from an Emax function with the free parameters Emax and ET50 for each patient. Emax and ET50 will be tested each per t-test for mean value differences between TAU + Placebo and TAU + Etifoxine.

Secondary endpoints: All secondary endpoints will be analyzed only exploratory and will be defined in detail in the Statistical Analysis Plan. There will be no multiplicity adjustments of the *p*-values.

Safety endpoints: All data concerning the safety of the investigational product will be listed and reported in comparison to placebo. All (serious) adverse events will be summarized in frequency tables and will further be reported separatly according to intensity and relation to the investigational product.

Imaging data will be analyzed using specialized software (FSL, http://fsl.fmrib.ox.ac.uk) and with own Matlab- and Python-based programs. In all conducted tests we make no assumptions on the distribution of the data and therefore use computer-based randomization tests for the evaluation of significance.

### Interim analyses {21b}

Not applicable; no interim analyses are planned.

### Methods for additional analyses (e.g., subgroup analyses) {20b}

Not applicable; no subgroup analyses are planned.

### Methods in analysis to handle protocol non-adherence and any statistical methods to handle missing data {20c}

The statistical analysis is performed using the intention-to-treat population. To check the robustness of the effects, further analysis will be performed on the per-protocol collective. We will perform a sensitivity analysis to examine the effect of missing data in primary and secondary outcome variables. Missing data will not be replaced within this clinical trial.

### Plans to give access to the full protocol, participant-level data and statistical code {31c}

The full protocol as well as datasets used and/or analyzed during the current study can be made available by the corresponding author upon reasonable request.

## Oversight and monitoring

### Composition of the coordinating center and trial steering committee {5d}

This is a single-centre study and the coordinating centre of this study is the Department of Psychiatry and Psychotherapy of the University Regensburg. Two study assistants and two study coordinators (one medical investigator and one clinical psychologist) conduct local organization including the identification of potential recruits. Consent consultations are held by a medical investigator or a clinical psychologist of the study team. There are weekly meetings between the Principle Investigator, its deputy, the study assistants, and the study coordinators as well as PhD students whot are involved in the clinical trial. The whole study is closely monitored by the Centre for Clinical Studies of the University Hospital Regensburg. There is no Trial Steering Committee and no Stakeholder and Public Involvement Group for this randomized clinical trial.

### Composition of the data monitoring committee, its role and reporting structure {21a}

Data management is carried out by independent data managers of the Center for Clinical Studies, University of Regensburg. The primary check of the study data (source data comparison) is carried out by clinical monitoring. The completeness and plausibility of the recorded study data is checked by the data management using computer-aided and manual data checks. In the event of necessary data clarifications (queries), these are transmitted electronically to the monitor and the study center, where they are corrected or supplemented in the database.

### Adverse event reporting and harms {22}

Adverse events (AE) are recorded from the time the informed consent form is signed until the end of the clinical trial. At each study visit, the patient will be asked if there have been any health problems since the last visit. All AEs will be appropriately recorded, whether or not they are thought to be related to the investigational product. AEs are followed up for a maximum of 28 days after the end of trial participation until the symptoms have subsided, deviating laboratory values have returned to baseline values or, in the investigator’s opinion, no further findings are to be expected. This applies regardless of the causal relationship between the AE and the investigational product.

Incidents that meet the criteria of a serious adverse event (SAE) are reported by the ward staff to a medical member of the trial group. The latter immediately fills out an SAE form and forwards it to the investigator or his deputy. The investigator assesses a possible connection with the investigational medicinal product and makes a note of this. He forwards the SAE report form to the sponsor within 24 h of becoming aware of it.

If there is a suspicion of a causal relationship with the investigational product, the sponsor of the clinical trial is obliged to inform BfArM and the ethics committee. The maximum period is 15 days; in the event of a life-threatening or fatal case, the maximum period is reduced to 7 days.

### Frequency and plans for auditing trial conduct {23}

Monitoring will be performed by a monitor of the Center for Clinical Studies, University of Regensburg, which will regularly visit the center depending on the patient recruitment. It takes place according to a clinical monitoring plan. The study center was initiated by an initiation meeting in which the background of the study as well as the relevant procedures and measurements (e.g. getting informed consent, study medication, emergency unblinding) were presented. Regular visits will take place after every ten patients are included. For the first two patients, a 100% data comparison of the source date and the eCRF takes place.

### Plans for communicating important protocol amendments to relevant parties (e.g., trial participants, ethical committees) {25}

Any change to the study procedures defined as a substantial amendment according to the regulations of Good Clinical Practice must be made in written form, stating the reasons for the change. They must be signed by the investigator and his deputy as well as by the sponsor. They will be submitted to the ethics committee and the National Institute for Pharmaceutical Security (BfArM) and are only implemented after their approval. Changes in the technical conduct of the study that do not have a direct impact on the safety of study participants can be submitted to the ethics committee as a technical note. Administrative changes in responsibilities (outside actual patient care) can be reported to the ethics committee as an administrative change.

### Dissemination plans {31a}

We will publish the results of this clinical trial in international peer-reviewed journals and present them at national and international congresses. Both positive and negative results will be reported. We will further report the results in databases (EU Clinical Trials Register; German Register of Clinical Studies) and will create a lay summary for distribution to all participating patients. Within all publications, data protection is ensured for all patient-related data.

## Discussion

We here describe the study protocol for a single-center, randomized, placebo-controlled clinical trial on the effects of the TSPO ligand etifoxine in the treatment of depressive disorders. The primary objective of the trial is to investigate the effects of add-on treatment with etifoxine on clinical symptoms in patients with severe unipolar/bipolar depressive disorder. Clinical symptoms will be assessed using the HAMD-21 up to day 15 after the start of the treatment.

In advance, we were aware of some challenges in conducting this trial, including mainly the recruitment of eligible patients and their continuance in the study. We have taken several precautions to address these challenges as we pointed out in sections {15} and {18b}. Overall, we try to realize inclusion to the trial close in time to inpatient admission to increase the chance that included patients stay at the hospital until the end of the trial 29 days after starting the study medication. In any case, the primary outcome is measured at a relatively early time point (2 weeks after the start of the study treatment). Overall, the study team has a good track record in enrolling and following participants in previous trials and there were no drop-outs of participants in former trials investigating the effects of etifoxine at the site [47–49].

The merit of this study is that it is the first proof-of-concept study on the TSPO ligand etifoxine in the treatment of depression, which may promote endogenous neurosteroid synthesis. With its favorable side effects profile, it could become a valuable compound, which might help in enhancing the effects of established antidepressant medication as an alternative to exogenously administered neurosteroids, e.g., zuranolone*.*

## Trial status

The start of the recruitment was in April 2023. Currently (13th of January 2024), we included 15 patients. With a planned number of 50 patients, the recruitment and treatment period is estimated to be about a maximum of 33 months (until January 2026). The current protocol is version 4 of 7–7-2023.

## Data Availability

The datasets used and/or analyzed during the current study are available from the corresponding author on reasonable request.

## Abbreviations

AE: Adverse event
BDI: Beck-Depression-Inventory
BENDEP-SRQ: Benzodiazepine Dependence Self-Report Questionnaire
BfArM: *Bundesinstitut für Arzneimittel und Medizinprodukte* (National Institute for Pharmaceutical Security)
BHWSS: Benzodiazepine Hypnotics Withdrawal Symptom Scale
BOLD: Blood oxygenation level dependent
CANTAB: Cambridge Neuropsychological Test Automated Battery
CAR: Cortisol Awakening Response
Emax: Maximum effect which can be expected from a drug
ET50: Time point at which 50% of the maximal effect has occurred
FMRI: Functional magnetic resonance imaging
FNA: Face-name-associations
HAMD-21: Hamilton Depression Scale
IIT: Investigator-initiated trial
MADRS: Montgomery–Åsberg Depression Rating Scale
M.I.N.I.: Mini-International Neuropsychiatric Interview
PET: Positron emission tomography
PHQ-9: Patient Health Questionnaire 9
RSA: Representational similarity analysis
SAE: Serious adverse event
SSS: Stanford Sleepiness Scale
TAU: Treatment as usual
TSPO: Translocator protein
VAS: Visual analog scales
WOCBP: Women of Childbearing Potential
